# A Whole Exon Screening-Based Score Model Predicts Prognosis and Immune Checkpoint Inhibitor Therapy Effects in Low-Grade Glioma

**DOI:** 10.3389/fimmu.2022.909189

**Published:** 2022-06-13

**Authors:** Cheng Luo, Songmao Wang, Wenjie Shan, Weijie Liao, Shikuan Zhang, Yanzhi Wang, Qilei Xin, Tingpeng Yang, Shaoliang Hu, Weidong Xie, Naihan Xu, Yaou Zhang

**Affiliations:** ^1^ China State Key Laboratory of Chemical Oncogenomics, Tsinghua Shenzhen International Graduate School, Shenzhen, China; ^2^ Department of Biomedical Engineering, Tsinghua University, Beijing, China; ^3^ Key Lab in Healthy Science and Technology of Shenzhen, Tsinghua Shenzhen International Graduate School, Shenzhen, China; ^4^ School of Life Sciences, Tsinghua University, Beijing, China; ^5^ Open Faculty for Innovation, Education, Science, Technology and Art, Tsinghua Shenzhen International Graduate School, Shenzhen, China; ^6^ Department of Chemical Engineering, Tsinghua University, Beijing, China; ^7^ Research and Development Department, Shenzhen Combined Biotech Co., Ltd, Shenzhen, China

**Keywords:** METTL7B, PD-L1, prognosis prediction, glioma, m6A (N6-methyladenose), RNA stability

## Abstract

**Objective:**

This study aims to identify prognostic factors for low-grade glioma (LGG) *via* different machine learning methods in the whole genome and to predict patient prognoses based on these factors. We verified the results through *in vitro* experiments to further screen new potential therapeutic targets.

**Method:**

A total of 940 glioma patients from The Cancer Genome Atlas (TCGA) and The Chinese Glioma Genome Atlas (CGGA) were included in this study. Two different feature extraction algorithms – LASSO and Random Forest (RF) – were used to jointly screen genes significantly related to the prognosis of patients. The risk signature was constructed based on these screening genes, and the K-M curve and ROC curve evaluated it. Furthermore, we discussed the differences between the high- and low-risk groups distinguished by the signature in detail, including differential gene expression (DEG), single-nucleotide polymorphism (SNP), copy number variation (CNV), immune infiltration, and immune checkpoint. Finally, we identified the function of a novel molecule, METTL7B, which was highly correlated with PD-L1 expression on tumor cell, as verified by *in vitro* experiments.

**Results:**

We constructed an accurate prediction model based on seven genes (AUC at 1, 3, 5 years= 0.91, 0.85, 0.74). Further analysis showed that extracellular matrix remodeling and cytokine and chemokine release were activated in the high-risk group. The proportion of multiple immune cell infiltration was upregulated, especially macrophages, accompanied by the high expression of most immune checkpoints. According to the *in vitro* experiment, we preliminarily speculate that METTL7B affects the stability of PD-L1 mRNA by participating in the modification of m6A.

**Conclusion:**

The seven gene signatures we constructed can predict the prognosis of patients and identify the potential benefits of immune checkpoint inhibitors (ICI) therapy for LGG. More importantly, METTL7B, one of the risk genes, is a crucial molecule that regulates PD-L1 and could be used as a new potential therapeutic target.

## Introduction

Central nervous system (CNS) malignant tumors have one of the worst prognoses among all cancers, and glioma is the most common primary tumor of the CNS, accounting for approximately 80% of malignant brain tumors (1, 2). At present, the clinical classification of gliomas still follows the histological diagnostic criteria proposed by the WHO in 2007 (3). This classification method has significant limitations (4). One reason is that subjective preference easily differentiates judgments based on tumor histology between observers (5). As a result, the survival time of a group of patients with glioma may vary from weeks to years. It is difficult to explain this difference based only on histological grade. Although diffuse low- and intermediate-grade gliomas collectively constitute low-grade gliomas (LGGs, WHO grades II and III), which are rarer than grade IV gliomas (GBM, glioblastoma) due to their highly invasive nature, complete neurosurgical resection is impossible, leading to recurrence and malignant progression, eventually progressing to glioblastoma (3, 6–8). Therefore, it is necessary to propose new detailed diagnostic criteria that integrate the molecular changes in glioma.

For the treatment of glioma, traditional surgical resection is difficult and the residual tumor cells will further deteriorate. Radiotherapy has also been associated with epilepsy and mild dementia (9). Given these limitations, immune checkpoint inhibitor (ICI) drugs have proven to be promising treatments (10). In a phase III clinical trial of glioblastoma, the overall response rate of patients to nivolumab (PD-1 monoclonal antibody) was only 8%, but the overall survival time doubled (11). Considering the good therapeutic effect and high medical cost of glioma, there is an urgent need for a valuable biomarker to predict the benefits of immunotherapy in patients with glioma.

In this study, we focused on all genes in the LGG transcriptome data. We tried to develop a prognostic marker of LGG that can predict the routine prognosis of patients and the potential benefits of immunotherapy. We found that the immune response, extracellular matrix remodeling, and cytokine release were accelerated in the high-risk groups. In addition, high-risk patients are accompanied by the upregulation of most immune checkpoints represented by PD-1/L1 and the increase in tumor mutation burden (TMB) and CNV, suggesting that patients may respond better to the ICI of PD-L1 (12–15). We proved this through the TIDE score (16). In short, the model can predict the prognosis of patients and determine the possible benefits of ICI treatment. Finally, we also found a new molecule, METTL7B, in glioma, which reduces the expression of PD-L1 in cells by inhibiting the stability of PD-L1 mRNA and lead to the apoptosis of co-cultured T cells.

## Methods

### Publicly Available mRNA Data and Immune Gene Sets

Data from two publicly available datasets were incorporated into our study. TCGA RNA-seq data (FPKM) of samples from patients with LGG (Illumina HiSeq 2000) were acquired from the Genomic Data Commons (GDC) (http://portal.gdc.cancer.gov). According to the whole survival time, age, radiotherapy status, and glioma grades, 420 patients were collected and randomly (in a 7:3 ratio) categorized as training set and internal validation set. 529 glioma data was downloaded with complete clinical data and molecular subtyping data (IDH1 mutation, 1p19q codeletion, and MGMT methylation) from Chinese Glioma Genome Atlas (CGGA) (http://www.cgga.org.cn) to serve as external validation sets.

### Construction and Verification of Multivariate Cox Signature

According to the mRNA expression of risk genes, a stepwise Cox proportional hazards regression model was used. RF realized by the R package “randomforestsrc”, the number of feature trees was 100, and the number of random splits was 1. LASSO realized by R package “glmnet”, and 100 times cross-validation was carried out. Signature genes were obtained by taking the intersection of these two gene lists. Risk score formula was calculated by taking into account the expression of signature genes and correlation estimated Cox regression coefficients: Risk score = (exp Gene1 * coef Gene1) + (exp Gene2 * coef Gene2) +… +(exp Gene7 * coef Gene7). Patients with LGG were classified into a high-risk or low-risk group by ranking the given risk score. The thresholds of high- and low-risk groups were selected through the “survminer” package. The R package “timeROC” was used to test the time-dependent receiver operating characteristic curve (ROC) (17). The difference of overall survival (OS) between two groups in the three cohorts was assessed using Kaplan–Meier method and the two-tailed log-rank test. A Cox proportional hazards regression model was used to identify independent prognostic factors.

### Construction and Validation of Multigene Containing Nomogram

Nomogram was used to predict the survival probability by specific clinical parameters (18). We constructed the nomogram containing the multigene signature and other independent prognostic factors. The nomogram was calibrated at 1-year, 3-years, and 5-years using the R package “rms”. Decision curve analysis (DCA) analysis was used to assess the clinical application benefits of the multigene panel in the TCGA set (19).

### Biological Process and Pathway Enrichment Analysis

Using the R package “DESeq2” (20), DEGs between high- and low-risk groups were identified. Then, using Gene Ontology (GO) and Kyoto Encyclopedia of Genes and Genomes (KEGG) enrichment analysis, different pathways and items were identified between the two risk groups. In addition, we used GSEA (21) to dynamically score different enrichment items in the high- and low-risk groups.

### Weighted Gene Co-Expression Network Analysis

The R package “WGCNA” (22) was used to perform weighted gene co-expression network analysis (WGCNA) using the TCGA LGG expression matrix (FPKM). To build a scale-free network and calculate the network topology matrix, the gene expression matrix is weighted by a soft threshold. We use a dimension reduction algorithm to visualize the network module composed of co-expressed genes in glioma samples after clustering with the dynamic cut tree algorithm and merging similar modules.

### Evaluation of Immune Microenvironment With CIBERSORT and ssGSEA

The LM22 signature matrix, which is included in CIBERSORT, was used to estimate the distribution of 22 immune cell types (23). We ran 1000 iterations in R studio using the script provided in this paper to assess the difference in 22 immune cell infiltrations between the high and low risk groups and displayed the results in heatmap. ssGSEA was realized through the R package “GSVA” according to the analysis process and method provided by the official instruction (24).

### Analysis of Gene Mutation and Copy Number Variation

The copy number variation data in the TCGA database was downloaded through the ‘TCGAbiolinks’ R package (25), and the risk score and CNV were integrated. snp6 grch38 annotation file was downloaded in TCGA and analyzed with GISTIC2.0 (26). Gene mutation data was also obtained from the TCGA database. The occurrence of mutation events was calculated and matched with the risk score. Finally, the ‘maftools’ R package was used for visualization (27).

### Cell Culture and Construction of Stable Cell Lines

U251 cells, A172 cells, and Jurkat cells were purchased from the National Collection of Authenticated Cell Cultures. U251 and A172 were grown in DMEM medium with 10% fetal bovine serum (Gibco, California, USA). Jurkat cells were cultured in RPMI 1640 Medium with 10% fetal bovine serum. All medium was supplemented with 10 U/ml of penicillin-streptomycin, and all cells were cultured in a 5% CO2 humidified incubator at 37°C. The control shRNA and Lentivirus-based LINC00472-targeting shRNA vectors were purchased from GENECHEM (Shanghai, China). U251 cells were transiently transfected with these vectors and screened by puromycin at a 2 mg/ml concentration to generate stable monoclonal cell lines.

### PCR and Real-Time Quantitative PCR Analysis

According to the manufacture’s protocol, the total RNA was isolated using AG RNAex Pro Reagent AG21101. Real-time quantitative PCR was performed using the TransScript All-in-One First-Strand cDNA Synthesis SuperMix for qPCR (One-Step gDNA Removal) (TRANS, AT341-01) and the PerfectStart SYBR Green qPCR SuperMix (TRANS, AT601-01). The primers used are listed in **Supplemental Table S1**, and all the levels of mRNAs were measured and normalized to β-actin.

### Western Blotting

Western blotting was performed as previously described (28). The antibodies used for western blotting include METTL7B (Abclonal, A7200), CD274 (Abcam, 243877), and β-actin (CST, 8480S).

### Coculture Study and Assessment of Apoptosis

To examine the effect of tumor cells on lymphocyte apoptosis, a total of 5×10^6^ U251 cells were cocultured with 5×10^5^ Jurkat leukemia T cells in 6-cm plates for 24 h. Jurkat cells were collected and washed three times with PBS diluted in annexin binding buffer. For each sample, 5 ml (2.5 mg/ml) annexin V–FITC and 5 ml (50 mg/ml) propidium iodide were added to the cell suspension and incubated for 15 min at room temperature (25°C) in the dark. The extent of apoptosis in Jurkat cells was determined by flow cytometry using FITC–annexin V.

### Total m6A Modification Level and RNA Stability of Cells

The total amount of m6A in total RNA was measured using the m6A RNA Methylation Assay Kit (Fluorometric) (Abcam, ab233491), following the manufacturer manual. For each sample, 200 ng of total RNA from U251 cells were used. For RNA stability detection, cells were cultured overnight and then treated with actinomycin D 10 mg/mL at 0, 2, 4 and 6 h before trypsinization collection. The total RNA was extracted by TRIzol. Quantitative RT-PCR was conducted to determine the relative level of indicated mRNA.

### Statistical Analysis

The R software (version 4.1.2) was used for the statistical analysis. Statistical analysis of cell and molecular biology experiments was performed using GraphPad Prism 8.0 version. “ggplot2”, “ggpubr”, “vioplot” were applied to visualize the results of data analysis. Wilcoxon Signed Rank test and Student’s t test were used for statistical analysis between two groups, while the Kruskal-Wallis test was applied for statistical tests of more than two groups. When p less than 0.05, we considered the difference to be statistically significant.

## Results

### Identification of Prognosis-Related Genes in Low-Grade Glioma

**Figure 1:**
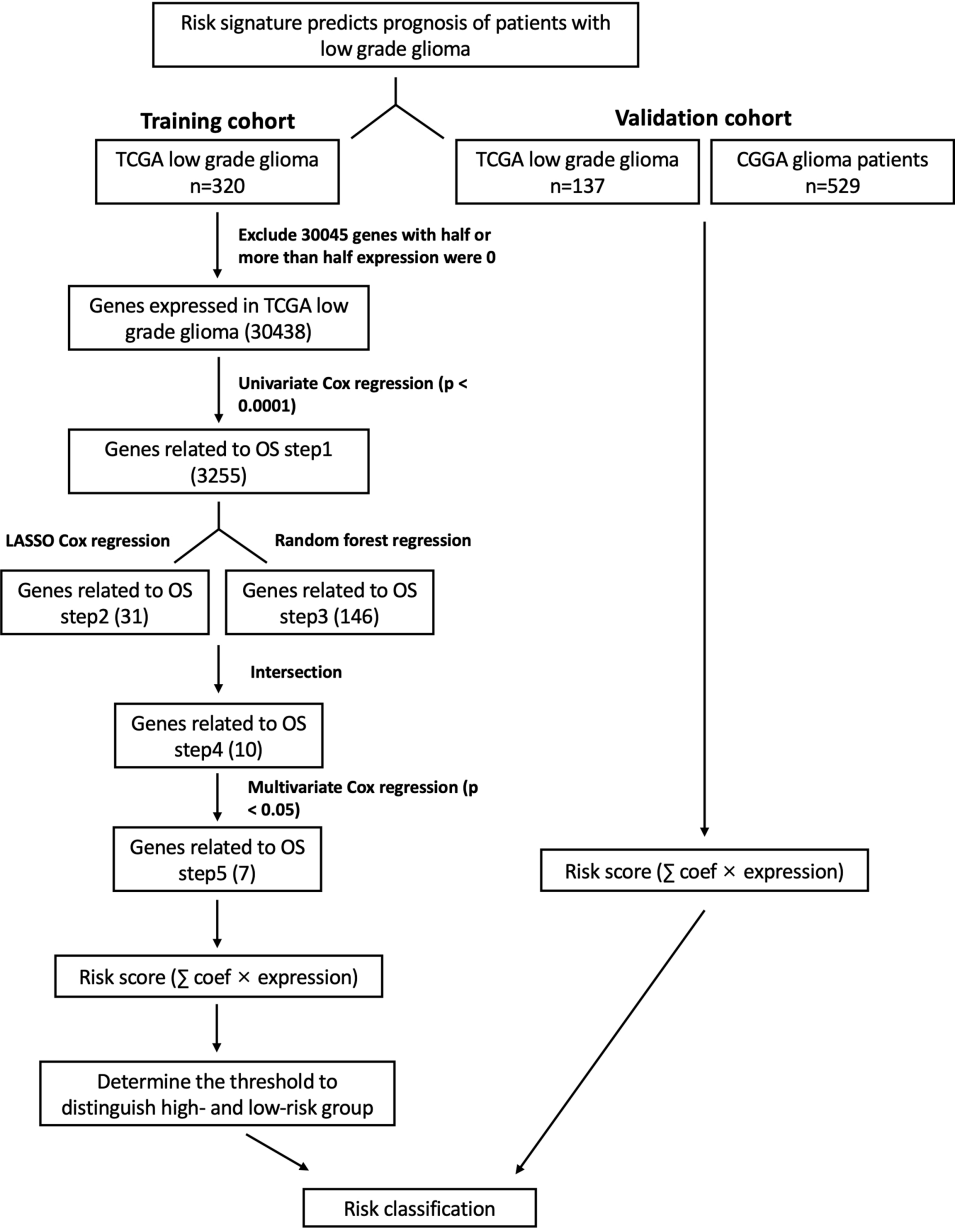
Schematic diagram of a gene screening strategy for prognosis prediction in this study.

In this study, 529 LGG samples were acquired from the TCGA database, and a total of 530 patients were obtained from the CGGA database. The glioma data from TCGA were randomly divided into a training set and a validation set at a ratio of 7:3. The process is shown in **Figure 1**; 32622 genes were selected because the gene expression level in half of the samples was more than zero. The expression of these genes in LGG samples was used for univariate Cox regression analysis. A total of 3432 genes were significantly (p < 0.001) associated with the overall survival of patients in the TCGA training set.

**Figure 2:**
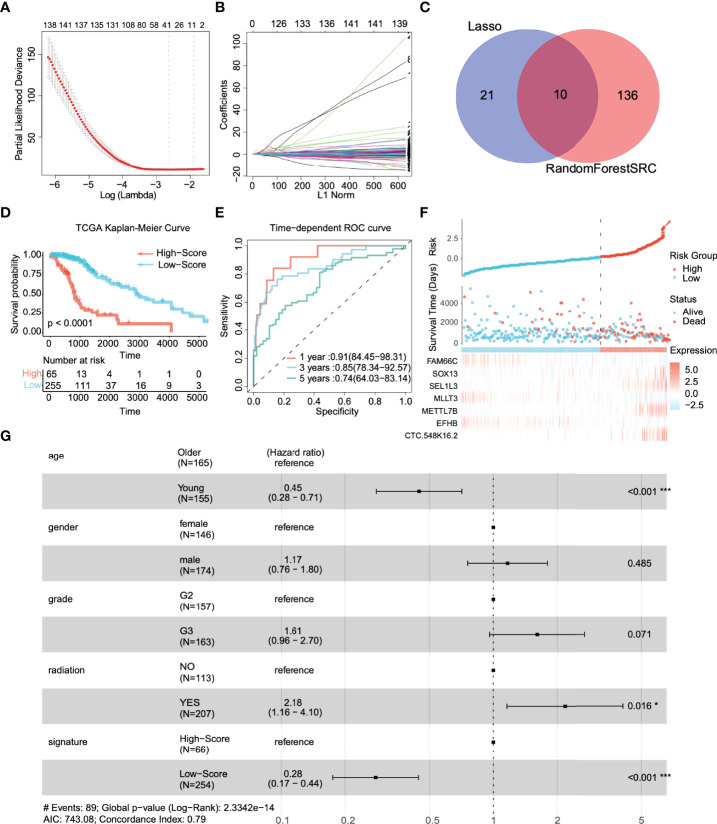
Identification of prognostic genes and survival prediction of patients with low-grade gliomas in the TCGA training cohort. **(A)** 100-fold cross-validation for tuning parameter selection in the LASSO model. **(B)** The distribution of regression coefficients of significantly related genes in the model. **(C)** Gene selection through two algorithms based on the Venn plot. **(D)** Kaplan–Meier curves of overall survival (OS) in low-grade glioma are based on the risk score. **(E)** Time-dependent ROC curve of the risk gene signature at 1, 3, and 5 years in the TCGA training cohort. **(F)** Distribution of risk score, survival time, and gene expression panel. **(G)** Subgroup analysis shows the effect of different clinical features in TCGA for OS patients with low-grade glioma. Hazard ratios with 95% confidence intervals are shown in each different group. *p < 0.05, ***p < 0.001.

We included these significant genes in LASSO and RF regression (**Figures 2A, B**), two algorithms screened 31 and 146 genes related to the clinical outcome of glioma patients, respectively. Then, we obtain the intersection of these two algorithms. **Figure 2C** shows that 10 genes existed simultaneously in the two regression analysis results. Multivariate Cox regression analysis was performed on these ten genes, and seven genes with P values < 0.05 were selected (**Table 1**).

**Table 1:** Multivariate Cox analysis was used to further screen prognostic factors and corresponding coefficients of the linear model.

Characteristics	Hazard Ratio	CI95	p.value	Coef.
ARL3	0.67	0.4-1.14	0.143	
CTC-548.K16.2	31.58	1.39-715.54	0.030	3.818
EFHB	0.60	0.37-0.98	0.040	-0.539
HILS1	1.12	0.56-2.23	0.741	
METTL7B	1.23	1.02-1.47	0.027	0.194
MLLT3	0.49	0.29-0.82	0.007	-0.813
RP11.893F2.14	2.33	0.84-6.47	0.106	
SEL1L3	1.41	1.16-1.72	0.001	0.466
SOX13	1.57	1.26-1.98	0.000	0.498
FAM66C	1.32	1.06-1.63	0.032	-0.216

### The 7-Gene Signature Can Accurately Predict the Prognosis of Patients With Low-Grade Glioma

According to the above feature selection algorithm, CTC-548K16.2, EFHB, METTL7B, MLLT3, SEL1L3, SOX13, and FAM66C were used to build a multigene signature for predicting the survival of LGG patients. The risk score of each patient was estimated based on the expression of these genes and their corresponding coefficients, which were obtained by multivariate Cox regression analysis. Patients were categorized into a significant risk group based on the optimized risk value based on the results of ROC analysis.

First, we investigated the performance of the multigene signature in predicting the OS of LGG patients. The K–M curve suggested that the clinical outcome was significantly worse in the high-risk group than in the low-risk group (p < 0.0001) (**Figure 2D**). Furthermore, the time-dependent ROC curve shown in **Figure 2E** shows that the multigene signature has excellent performance in predicting survival events (the areas under the curves (AUC) at one year, three years, and five years were 0.91, 0.85, and 0.74, respectively). **Figure 2F** illustrates the risk score distribution of patients, the survival time, and the heatmap of the seven gene expression profiles in each patient. In short, the risk score proved to be highly significant for patients with glioma in the training set.

We identified clinically independent prognostic factors. As shown in **Figure 2G**, young age (HR = 0.45, 95% CI: 0.28~0.71, p < 0.001) and low-risk score (HR = 0.28, 95% CI: 0.17~0.44, p < 0.001) were protective factors, and glioma grade (HR = 1.61, 95% CI: 0.96~2.70, p = 0.071) and radiotherapy (HR = 2.18, 95% CI: 1.16~4.10, p = 0.016) seemed to be risk.

### The 7-Gene Signature Still Has Good Performance in the Internal and External Validation Sets

**Figure 3:**
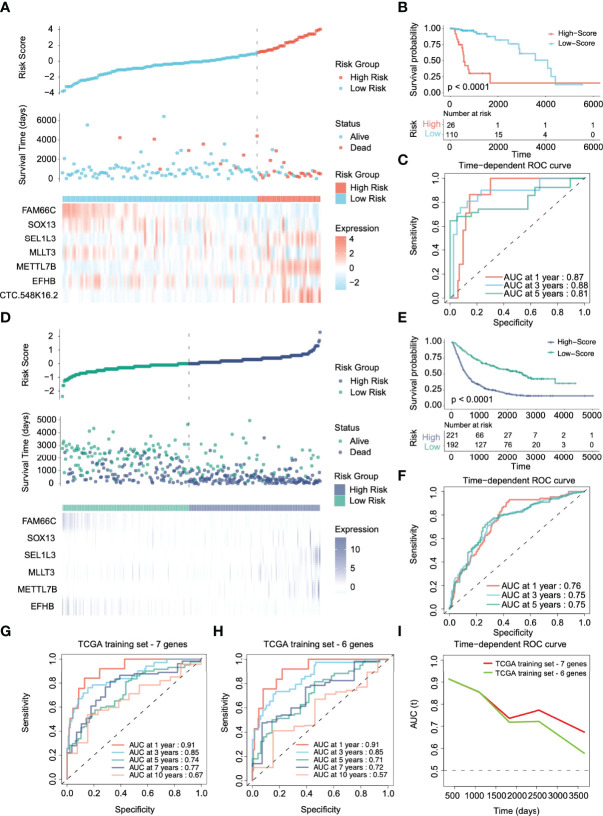
Validation of the prognostic performance of the risk stratification gene signature in TCGA and CGGA cohorts. **(A, D)** Distribution of risk score, survival time, and gene expression panel in TCGA and CGGA validation cohorts. **(B, E)** Kaplan–Meier curves of OS based on the risk score in the TCGA and CGGA cohorts. **(C, F)** ROC curve of the risk gene signature at 1, 3, and 5 years in the TCGA and CGGA cohorts. **(G, H)** Time-dependent ROC curve of the risk gene signature at 1, 3, 5, 7, and 10 years in the TCGA cohort with and without CTC-548K16.2. **(I)** The area under the ROC curve (AUC) of prognosis prediction using different risk stratification models in the TCGA dataset.

To validate the performance forecast of the multigene signature, we used glioma patient data from TCGA and CGGA as the internal verification and external verification cohort, respectively. With the same coefficients, patients were divided into a high-risk group (N=26 in TCGA, N=221 in CGGA) and a low-risk group (N=110 in TCGA, N=192 in CGGA) based on the expression of 7 signature genes (**Figures 3A, D**). In the internal verification cohort of TCGA, K–M survival analyses showed that patients in the low-risk group had significantly better OS than those in the high-risk group (**Figure 3B**, p < 0.0001). The time-dependent ROC curve revealed that for predicting prognosis at 1, 3, and 5 years, the AUCs were 0.87, 0.88, and 0.81, respectively (**Figure 3C**). Because the CGGA dataset still contains grade IV gliomas, it suggests that the signature also has potential application value in high-grade gliomas.

However, in the CGGA external verification cohort, CTC-548K16.2 was removed due to probe loss. We used the same coefficient to integrate other genes for analysis. The 6-gene signature in the CGGA external verification set also had a superior ability to distinguish the clinical outcomes of the high-risk group and the low-risk group (**Figure 3E**, p < 0.0001). The 1-, 3-, and 5-year AUCs of the time-dependent ROC curves were 0.76, 0.75, and 0.75, respectively (**Figure 3F**). The results showed that although one variable was removed, the multigene prediction model still had good differentiation for the prognosis of glioma patients.

CTC-548K16.2 is a noncoding RNA that has low expression in the TCGA cohort. Next, we evaluated the significance of CTC-548K16.2 in the prediction signature. In the TCGA dataset, the AUC of the seven-gene model considering CTC-548K16.2 was 0.91 (1 year), 0.85 (3 years), 0.74 (5 years), 0.77 (7 years), and 0.67 (10 years) (**Figure 3G**). With the increase in survival time, the AUC showed a downward tendency. However, the 1-, 3-, 5-, 7-, and 10-year AUCs were 0.91, 0.85, 0.71, 0.72, and 0.57, respectively, when the 6-gene signature was used for prediction (**Figure 3H**). **Figure 3I** shows that the AUC decreased sharply at approximately 1800 days. These results suggest that CTC-548K16.2 may be related to the late prognosis of glioma patients.

### The Signature Has Suitable Identification for Different Clinical Subgroups and Molecular Subgroups

We aimed to determine whether the signature has universal applicability, whether it has a more accurate prediction for different types of patients, such as age or gender, and whether is unsuitable for a specific type of patient. We divided the patients into different subgroups according to clinical information. For all subgroups in the internal training set, the OS time of high-risk patients was shorter than that of low-risk patients (**Figure S1**). Signatures can achieve satisfactory identification in most subgroups in the validation set, except for G2 gliomas (**Figure S2**). One reason may be that the prognosis of G2 is usually good, so the number of samples identified as high-risk is minimal (only 3 patients in the high-risk group).

In terms of molecular subgroups, the IDH1 wild-type group (76%) had a higher proportion of high-risk patients than the IDH1 mutant group (34%) (**Figure S3A**). In addition, the percentage of high-risk patients in the 1p19q codeletion group (33%) was lower than that in the nondeletion group (58%) (**Figure S3B**). There was no significant difference in MGMT promoter methylation (**Figure S3C**). Regarding different molecular characteristics, the OS time of the high-risk group was significantly shorter (**Figures S3D-I**).

### The Nomogram Integrated Signature Shows That the Clinical Benefit to Patients Has Been Improved

**Figure 4:**
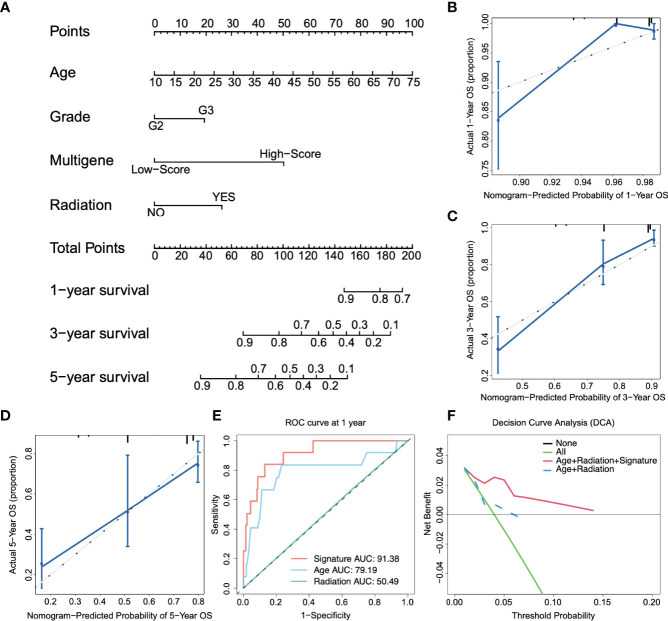
Construction of the nomogram. **(A)** Nomogram predicts 1-, 3-, and 5-year OS for low-grade glioma patients based on the risk signature and other clinicopathological parameters. **(B–D)** The calibration curves of the nomogram for predicting and observing 1-, 3- and 5-year OS. **(E)** ROC curve of the risk gene signature and other parameters at one year. **(F)** The decision curve analysis (DCA) shows the clinical benefits of patients after risk stratification.

A prognostic nomogram is a quantitative method for clinicians to predict the survival of LGG patients (29). We integrated clinically independent prognostic factors that were identified before. Nomogram was constructed based on these factors to predict the 1-year, 3-year, and 5-year survival probability of glioma patients (**Figure 4A**). The calibration plot closely resembled the ideal diagonal curve at 1-year, 3-year, and 5-year (**Figures 4B–D**), and the C-index of the nomogram was 0.807, suggesting that the performance of the nomogram was reliable.

**Figure 4E** shows that the AUC of the 1-year ROC curve was significantly higher than that of age (AUC = 0.791) and radiation (AUC = 0.505). In addition, we included independent prognostic factors to compare the decision curve analysis (DCA) of the predictive models with or without risk scores. We found that the clinical benefit of patients with risk score integration was much greater than that of patients with only age, grade, and other factor integration (**Figure 4F**). In other words, compared to the conventional clinical classification system, the nomogram with the risk score had a better performance in predicting survival outcomes. Therefore, these results illustrated that the nomogram could be used to predict the prognosis of glioma patients in clinical practice.

### Immune Response and Extracellular Matrix Remodeling Were Significantly Activated in High-Risk Patients

**Figure 5:**
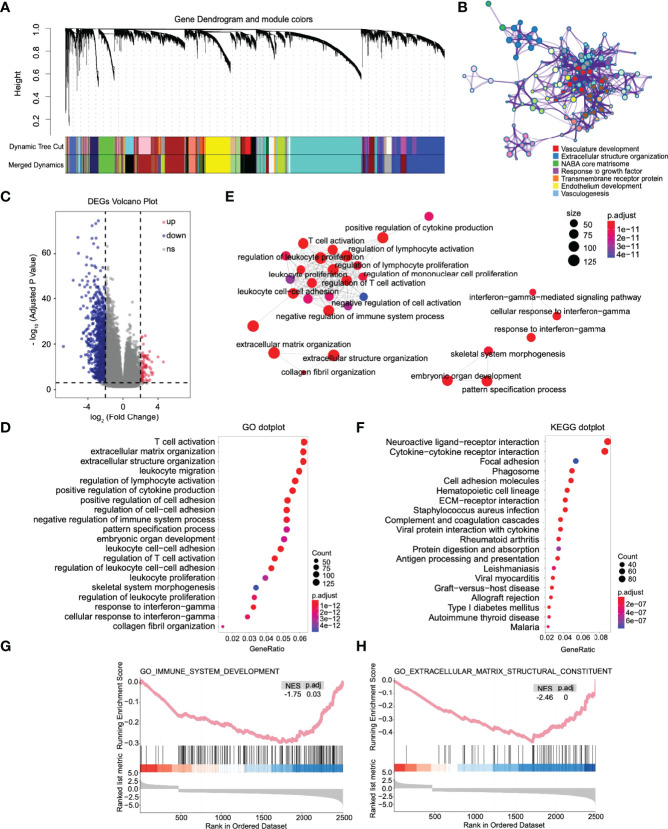
The immune response, extracellular matrix remodeling, and other pathways were significantly activated in high-risk patients. **(A)** Combining the modules with slight dissimilarity, 19 weighted gene coexpression subnetworks were obtained by a dynamic tree cut algorithm. **(B)** GO enrichment analysis was performed on all genes in the coexpression module where mettl7b is located. **(C)** Volcano plot of differentially expressed genes between high- and low-risk patients. **(D)** Dot plot of Gene Ontology (GO) enriched terms colored by p values. **(E)** Significant GO items were clustered according to biological function. **(F)** Dot plot of Kyoto Encyclopedia of Genes and Genomes (KEGG) enriched terms colored by p values. Gene set enrichment analysis between high- and low-risk patients. There was a significant enrichment of immune **(G)** and extracellular matrix remodeling **(H)** in the high-risk group. NES, normalized enrichment score.

To further determine the functional position of risk stratification genes in glioma progression. We selected 20000 genes according to the median absolute deviation and transformed glioma expression profiles (TCGA) into gene co-expression networks using the WGCNA package, as described previously (22). The soft threshold (beta = 4) was selected to build a scale-free network and check the mean connectivity of the network (**Figure S4A**). **Figure S4B** is used to verify the network node connection statistics and scale-free distribution. The fractional-step algorithm constructs the modules and calculates the correlation (**Figure S4C**). Then, the clusters with a degree of difference less than 0.2 were merged, and 19 different co-expression modules were finally obtained (**Figure 5A**).

Through the enrichment analysis of the WGCNA co-expression modules, we found that the risk stratification genes were located in four different co-expression modules. MLLT3 and FAM66C are light green in the network, and SEL1L3 and SOX13 are turquoise. The genes in these two modules are mainly related to the nervous system and synapses (**Figures S5A, B**). EFHB, which is depicted in brown, is primarily involved in DNA metabolism and chromatin remodeling in gliomas (**Figure S5C**). Finally, METTL7B, depicted in royal blue, is engaged mainly in vasculogenesis and extracellular structure organization (**Figure 5B**), suggesting that METTL7B has the closest relationship with glioma invasion, migration, and vasculogenesis among the risk stratification genes.

Then, based on the DESeq2 algorithm (20), we analyzed the differentially expressed genes between the high- and low-risk groups from the TCGA cohort, including 3883 upregulated and 1101 downregulated genes. The log2 enrichment ratio and -log10 adjusted p were visualized in a volcano plot (**Figure 5C**). GO analysis indicated that these genes could be categorized into inflammatory signaling pathways and immune responses, including T-cell activation and leukocyte and lymphocyte activation (**Figure 5D**). GO items with statistical significance were mainly concentrated in three clusters: immune response, extracellular matrix remodeling, and interferon-gamma mediated immune response (**Figure 5E**). KEGG analysis showed that the DEGs were mainly associated with essential biological processes, including ECM-receptor interactions, phagosomes, focal adhesion, the JAK-STAT signaling pathway, and the cAMP signaling pathway (**Figure 5F**). Fold changes in the mRNA expression levels of DEGs between the high- and low-risk groups were calculated and preranked in GSEA, and it revealed that the low-risk group was significantly associated with immune system development (NES = -1.75, p.adj = 0.03, **Figure 5G**) and extracellular matrix structural constituents (NES=-2.46, p.adj < 0.0001, **Figure 5H**).

### High-Risk Patients Have Prominent Immune Cell Infiltration and Increased Expression of Immune Checkpoints

**Figure 6:**
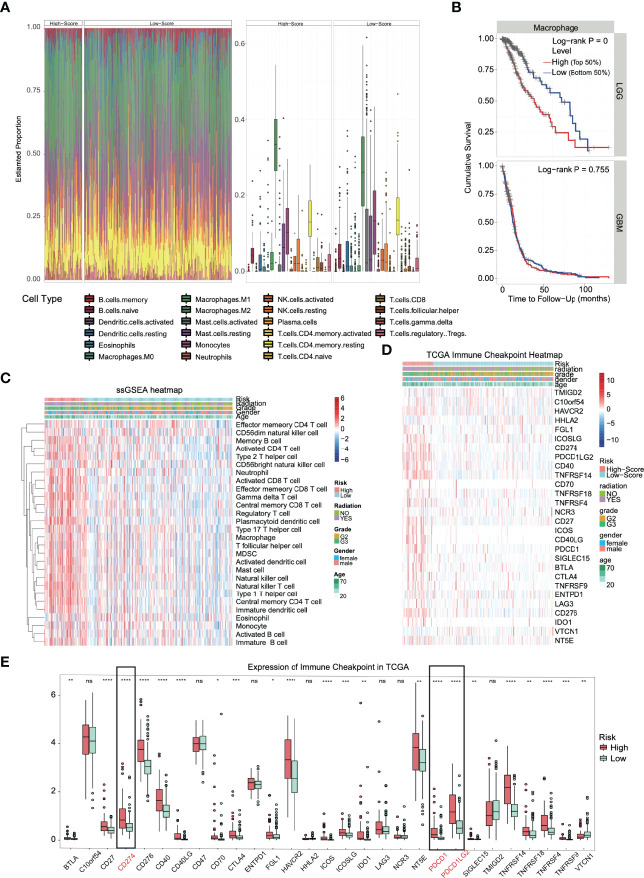
Immune infiltration and immune checkpoint expression in the high-risk group. **(A)** Heatmap and boxplot of the CIBERSORT algorithm evaluating the proportion of 22 kinds of immune cell infiltration in high- and low-risk solid tumors. **(B)** The proportion of macrophage infiltration was significantly correlated with the overall survival time of patients with low-grade glioma. **(C)** Heatmap of the immune cell infiltration landscape in high- and low-risk solid tumors using the ssGSEA algorithm. **(D)** The heatmap integrated the clinical features and the expression of the immune checkpoint in the TCGA dataset. **(E)** In TCGA datasets, most of the immune checkpoints were activated in high-risk groups. *p < 0.05, **p < 0.01, ***p < 0.001, ****p < 0.0001, ns. (no significance).

Functional enrichment analysis (GO, KEGG and GSEA) found immune response activation in high-risk patients, and we further analyzed this difference. We conducted different machine learning approaches to integrate multidimensional immune-related variables for every patient. CIBERSORT used a linear model to predict the content of immune cells in the tumor microenvironment and evaluated the accuracy of the results by 1000 permutation tests. In **Figure 6A**, patients in the high-risk group had a significantly higher proportion of CD8+ T cell, M1, and M2 types of macrophages and a substantially lower proportion of activated mast cells. The increased ratio of macrophage infiltration is associated with a worse prognosis in LGG, which seems to be unrelated to patients with glioblastoma (**Figure 6B**). We also conducted ssGSEA to evaluate the association with immune-infiltrating cells and the gene signature in individual glioma samples; however, the two algorithms are different. We obtained similar conclusions, and ssGSEA indicates that we should pay more attention to the general upregulation of the proportion of immune cell infiltration (**Figure 6C**).

The huge difference in the immune landscape suggests that high-risk patients may have different benefits from immunotherapy. Therefore, we expanded our analysis to 28 immune checkpoint molecules, including the B7-CD28 family (30, 31), TNF superfamily (32), and others (33–35). Surprisingly, most immune checkpoints were upregulated (**Figure 6D**), including the B7-CD28 family (p < 0.0001: CD274, CD276, ICOS, PDCD1 and PDCD1LG2), TNF superfamily (p < 0.0001: CD40, CD40LG and TNFRSF14) and others (p < 0.0001: HAVCR2) (**Figure 6E**). Based on the outstanding performance in the therapeutic effect of PD-1/L1 inhibitors, PD-L1 and PD-1 in glioma deserve more attention (36).

### The Amplification of TMB and CNV in the High-Risk Group Confirmed the High Response of ICI Therapy

We found that the majority of immune checkpoints in patients in high-risk group were upregulated, especially PD-1/L1. The high expression of these molecules has been proven to be related to patients’ better response to ICI therapy. In addition to the detection of PD-1/L1 molecules, the increase in TMB has also been proven to be related to the effectiveness of immunotherapy and TMB detection has been used as a clinical reference guide (12, 13). Therefore, we analyzed gene mutation and CNV, hoping to support our view further.

**Figure 7:**
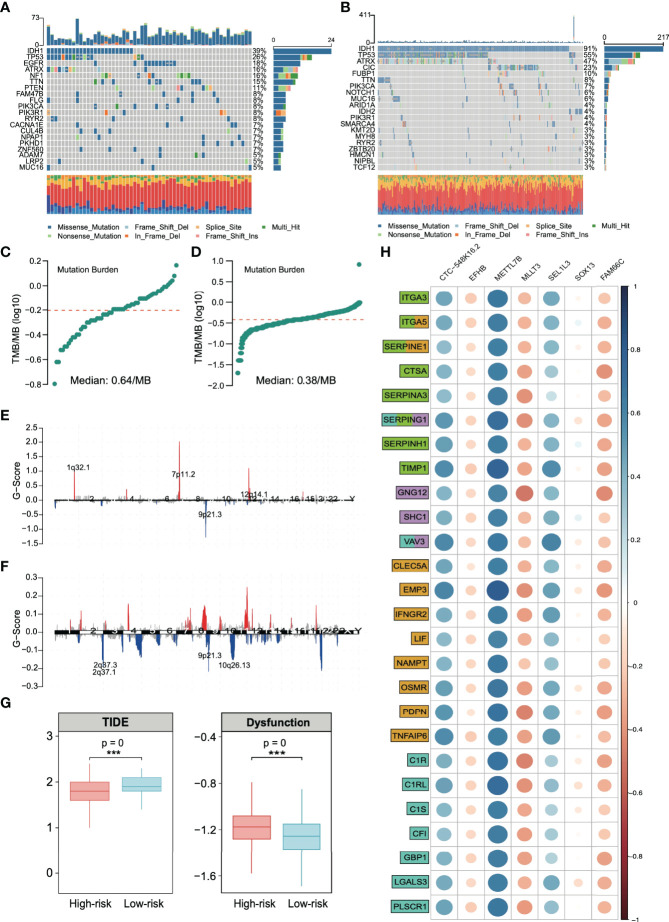
Analysis of single-nucleotide polymorphisms (SNPs) and copy number variations (CNVs) in patients in the high- and low-risk groups. Single-nucleotide polymorphisms in patients in the **(A)** high- and **(B)** low-risk groups. Tumor mutation load in patients in the **(C)** high- and **(D)** low-risk groups. Copy number variation in patients in the **(E)** high- and **(F)** low-risk groups. **(G)** Based on the calculation of TIDE, the prediction score of each sample (left) the difference of T-cell dysfunction and (right) the difference of TIDE score between high-risk and low-risk groups. ***p < 0.001. **(H)** Spearman correlation between risk stratification genes and extracellular matrix remodeling markers (green), chemokines (lilac), inflammatory factors (yellow), and immune activation markers (cyan). Gene function annotation is from the MSigDB database.

The analysis of SNP in the high- and low-risk groups (**Figures 7A, B**, respectively) revealed that IDH1 and TP53 were in the top two with the highest frequency of gene mutation. Among them, there were 39% IDH1 mutations in the high-risk group and 91% in the low-risk group, which is consistent with previous reports that IDH1 wild-type gliomas tend to have a significantly worse prognosis (37). We analyzed the TMB (the frequency of mutation events per million bases). The results showed that the average TMB of high-risk patients was 0.64/MB (**Figure 7C**), and that of low-risk patients was 0.38/MB (**Figure 7D**), which was nearly twice the difference. CNV analysis also showed significant copy number amplification in the high-risk group, which mainly involved three regions (1q32.1, 7p11.2, and 12q14.1, **Figures 7E, F**). All these conclusions confirm that the high-risk group may have higher benefits from immunotherapy. The TIDE score was used to test our conclusion. A higher TIDE score means that patients are unlikely to benefit from immunotherapy (16). We found that the TIDE score in the high-risk group was low, suggesting more significant benefits of immunotherapy. We also observed a higher proportion of T-cell inactivation, indicating the existence of immune escape (**Figure 7G**).

By integrating all the current conclusions, we returned to the risk grading gene itself and selected genes related to extracellular matrix (KEGG_ECM_RECEPTOR_INTERACTION, NABA_ECM_REGULATORS, and REACTOME_ECM_PROTEDGLYCANS, total of 366 genes), chemokines (KEGG_CHEMOKINE_SIGNALING_PATHWAY and WP_CHEMOKINE_SIGNALING_PATHWAY, total of 354 genes), inflammatory factors (HALLMARK_INFLAMMATORT_RESPONSE, total of 200 genes) and immune activation (GO_BP_ACTIVATION_OF_IMMUNE_RESPONSE, total of 563 genes) from the MsigDB database (https://www.gsea-msigdb.org/gsea/msigdb/) to analyze the relationship of signature genes and these signaling pathways.

We found that the extracellular matrix and immune activation-related genes, chemokines, and inflammatory factors were significantly activated in high-risk solid tumors (**Figure S6**). These results confirm that the signature genes regulate a molecular signaling network in glioma, which is strongly associated with tumor extracellular matrix remodeling and immune response. **Figure 7H** shows that METTL7B has the closest relationship with these immune response-related genes among the risk stratification genes. Additionally, METTL7B has the highest correlation coefficient.

### Knockdown of METTL7B in Glioma Promotes the Expression of PD-L1

At present, there is no report on the definite function of METTL7B in glioma, especially its relationship with the immune response. Considering the expression difference between the two risk groups in **Figure 6E**, we analyzed PD-L1, which is mainly expressed on the side of tumor cells and has been widely developed as a target of tumor immunotherapy. The relationship between this molecule and METTL7B was studied *in vitro*.

**Figure 8:**
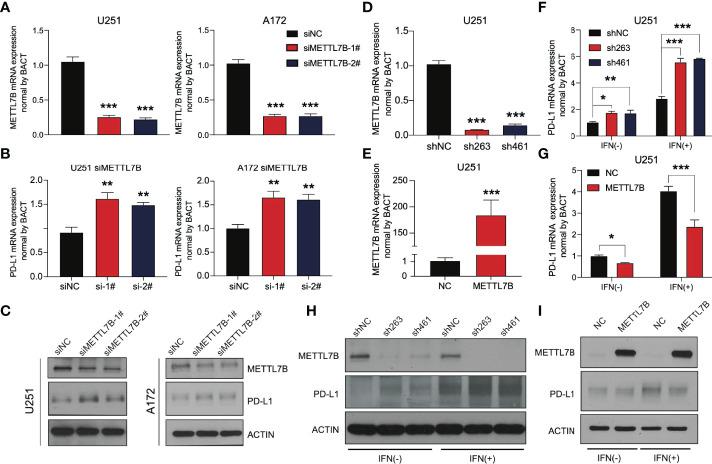
METTL7B affects the expression of PD-L1 in glioma. qRT–PCR showed that the METTL7B siRNAs in **(A)** U251 and **(B)** A172 cells could significantly downregulate METTL7B at the transcriptional level. **(C)** The expression of METTL7B and PD-L1 in U251 siMETTL7B and A172 siMETTL7B cells was detected by Western blot. **(D)** The expression of METTL7B mRNA in the U251 shMETTL7B cell line was detected by qRT–PCR. **(E)** The expression of METTL7B mRNA in U251 METTL7B-overexpressing cells was detected by qRT–PCR. The expression of PD-L1 in **(F)** U251 shMETTL7B and **(G)** overexpression cell lines in the presence and absence of interferon-gamma (80 ng/mL) was detected by qRT–PCR. The expression of PD-L1 in **(H)** U251 shMETTL7B and **(I)** overexpression cell lines in the presence and absence of interferon-gamma (80 ng/mL) was detected by Western blot. *p < 0.05, **p < 0.01, ***p < 0.001.

We selected the human glioma cell line U251 and glioblastoma cell line A172 to realize the knockdown of METTL7B through small interfering RNA (siRNA), as shown in **Figure 8A**. qRT–PCR showed that the two siMETTL7B targets could achieve a specific inhibition efficiency. We detected the expression of PD-L1 in two cell lines transfected with siMETTL7B and the results showed that the transient inhibition of METTL7B increased the mRNA level of PD-L1 (**Figure 8B**). We also verified this conclusion by Western blotting (**Figure 8C**). To further confirm this change, we transfected shRNA with lentivirus and transfected exogenous plasmids to knock out and overexpress METTL7B (**Figures 8D, E**). Consistent with the above conclusions, knockout of METTL7B further increased the expression of PD-L1, and overexpression reduced the level of PD-L1 (**Figures 8F, G**). Interferon-gamma is usually produced by activated T cells and NK cells and is responsible for inhibiting the growth of tumor cells (38). We used interferon-gamma to simulate the immune process of the body. We found that interferon-gamma can make this change more significant, and the Western blot results obtained a consistent conclusion (**Figures 8H, I**).

### METTL7B Inhibits the Stability of PD-L1 mRNA, and This May Involve m6A Modification

**Figure 9:**
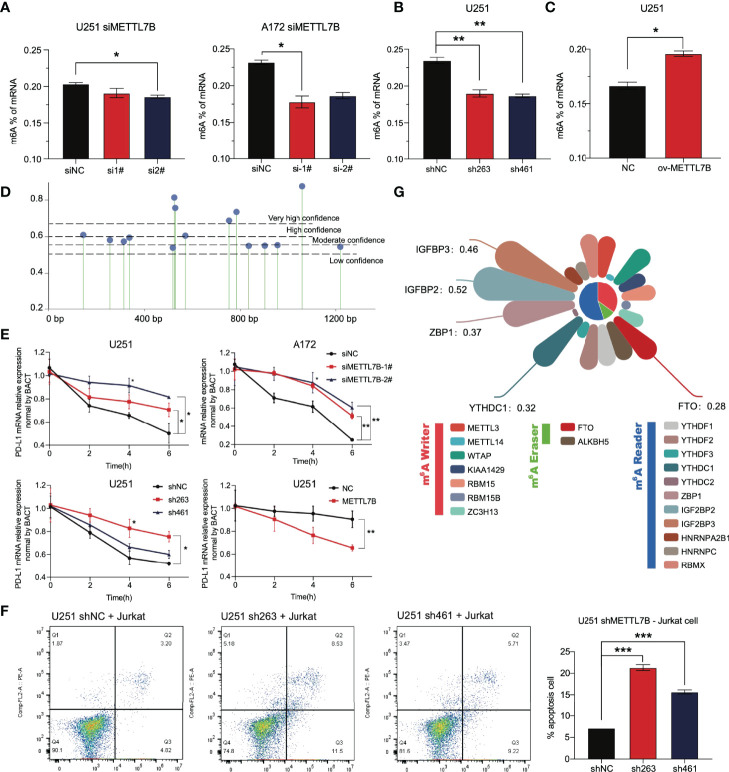
The change in METTL7B in glioma through m6A modification affects the stability of PD-L1 mRNA. **(A)** In U251 and A172 cells transfected with siMETTL7B, the total level of m6A modification decreased significantly. **(B)** In the U251 shMETTL7B cell line, the whole level of m6A modification decreased significantly. **(C)** In U251 cells overexpressing METTL7B, the total level of m6A increased significantly. **(D)** The potential m6A modification sites on PD-L1 mRNA molecules were predicted based on SRAMP. **(E)** Actinomycin D (10 µg/mL) inhibited the transcription of nascent RNA in different treatment groups. Cells were collected at 0 h, two h, four h, and six h. The PD-L1 mRNA content in cells was detected by qRT–PCR. **(F)** The U251 shMETTL7B cell line was incubated with suspended Jurkat lymphocytes for 24 hours (10:1), and the supernatant was collected to detect lymphocyte apoptosis. **(G)** Correlation coefficient between METTL7B and RNA m6A modification-related molecules in low-grade gliomas. *p < 0.05, **p < 0.01, ***p < 0.001.

At present, there are few reports on METTL7B, and there is no thorough report on its role in glioma. We analyzed other family members and found that the three members METTL3, METTL14, and METTL16 of the family have been reported to play the role of m6A writers and play vital roles in the occurrence and development of various tumors (39, 40). Considering an *in vitro* experiment by Franjic et al., it was verified that METTL7B functions as a methyltransferase *via* S-adenosylmethionine (SAM) as a methyl donor (41). We tried to study the relationship between METTL7B and intracellular m6A modification. The results showed that siMETTL7B reduced the overall m6A change in cells (**Figure 9A**), which seemed more evident in the shMETTL7B cell line (**Figure 9B**). Moreover, an increase in m6A modification was observed in overexpressing cells (**Figure 9C**). We predicted the posttranscriptional modification of RNA by the SRAMP method (42) and found 15 possible m6A modification sites on PD-L1 mRNA (**Figure 9D**). One of the most direct consequences of modifying intracellular RNA m6A is the change in the stability of the modified RNA. We verified that METTL7B inhibited the stability of PD-L1 mRNA. As shown in **Figure 9E**, knockdown of METTL7B in two different glioma cell lines increased the level of PD-L1 mRNA but decreased it in overexpressed cells. All these results suggest that the changes in total m6A modification in cells caused by METTL7B changes may affect the stability of PD-L1 mRNA molecules.

The PD-L1 molecule expressed on the surface of tumor cells binds to the PD-1 receptor on the surface of T lymphocytes, inhibits the function of T lymphocytes, and induces lymphocyte apoptosis. We cocultured Jurkat lymphocytes and U251 glioma cells to simulate the immune process of the body. The results showed that shMETTL7B cells increased the apoptosis of Jurkat lymphocytes coincubated (**Figure 9F**).

All the results show that METTL7B may regulate the expression of PD-L1 molecules through m6A, and m6A modification is likely to be an essential method for METTL7B to play a role in glioma cells, which has not been fully demonstrated at present. In glioma, we analyzed the correlation between METTL7B and the widely reported m6A writer, m6A eraser, and m6A reader. As we speculated, METTL7B has a significant correlation with many molecules, such as ZBP1, IGFBP2, and IGFBP3 (**Figure 9G**).

## Discussion

At present, many studies on the prognostic prediction of glioma patients have been reported. Most studies are based on predefined gene sets to screen prognostic factors. A very detailed analysis based on 24 autophagy characteristic genes constructed an accurate model to predict the prognosis of glioma patients (AUC of 3 years is 0.795) (43). Another study also accurately predicted the prognosis of glioma patients by integrating the expression of 19 lncRNAs related to hypoxia (AUC of 0.862 in one year) (44). We obtained much inspiration from these analyses. The risk prediction model based on a specific gene set can distinguish the prognosis of patients to a certain extent. However, the occurrence and development of the tumor is a highly complex biological process involving a variety of regulatory pathways. We believe that it is not comprehensive to predict only from several aspects of tumor cell development. Given this, we selected the most significant gene as the predictor through various machine learning screening methods starting from the whole genome. We analyzed the tumor as a whole, which achieved an excellent prediction effect (AUC of 0.91 in one year), showing the feasibility of the screening strategy and providing a reference for the development of subsequent cancer prognosis models.

Among these risk genes, the specific biological functions of SEL1L3 and CTC548K16.2 are rarely reported. FAM66C is a long noncoding RNA that has been found to regulate glycolysis and inhibit the proliferation and migration of tumor cells (45, 46). Consistent with these conclusions, we found that the expression of FAM66C in high-risk patients was significantly lower than that in low-risk patients, suggesting that FAM66C is more likely to play the role of a tumor suppressor gene in tumor cells and prevent tumor progression. As a widely reported transcription factor, SOX13 affects cell migration, invasion, and angiogenesis in various cancers and plays a role in an oncogene (47–49). This is closely related to the activation of extracellular matrix remodeling in high-risk people found in our study. For the EFHB gene, Takaoka et al. found through complete exon sequence analysis that EFHB single-nucleotide variation may induce the accumulation of DNA double-strand breaks in human AML cells (50). Similarly, we found that the coexpression subnetwork of EFHB in glioma regulates DNA metabolism and chromatin remodeling of tumor cells. In addition, MLLT3, as a developmental active epigenetic modifier during the generation of cortical projection neurons, participates in the development of the cerebral cortex by regulating the methylation of histone H3K79 (51). The WGCNA part of this study identified this gene as a factor participant in the development of the nervous system, neuronal differentiation, and ion transport. These results suggest the potential function of MLLT3 in glioma. Most studies on the biological function of risk grading genes are consistent with our results in glioma. These results also suggest that the above genes may have similar roles in glioma.

For predicting whether patients can benefit from PD-1/PD-L1 inhibitor therapy, at present, the detection of PD-L1 has been proven to be an effective method, and the conclusion that patients with high expression of PD-L1 have better overall survival and remission rates after receiving immunotherapy has been widely recognized. In addition to detecting the expression level of PD-L1 at the genomic level, it has also been confirmed that the higher the TMB is, the higher the efficacy of immunotherapy, and the detection of TMB has been written into the guidance guidelines of multiple clinical immunotherapy trials (52, 53). In addition to these two, a recent study also reported that CNV of plasma cell-free DNA in blood could predict the clinical results of PD-1 inhibitors combined with lenvatinib and other ICI-based hepatobiliary cancer treatments (15). In this study, we found that in the high-risk patients identified by the signature, the expression of PD-L1 increased significantly, accompanied by an increase in TMB and CNV, indicating the high potential benefits of PD-L1 ICI therapy in high-risk patients in all aspects. Therefore, this signature can accurately predict the prognosis of glioma patients and help identify the benefits of ICI therapy. We verified our conclusion through the TIDE score.

We identified a new functional molecule, METTL7B, for the first time in an *in vitro* experiment in a risk grading gene. We found that knockdown of METTL7B leads to increase in PD-L1, and high expression of PD-L1 is often accompanied by immune escape and malignant progression of the tumor. Recently, Song et al. reported that METTL7B in lung adenocarcinoma reversed resistance to epidermal growth factor receptor (EGFR)-tyrosine kinase inhibitors by changing m6A modification (54). As a new molecule in glioma, we demonstrated that METTL7B participates in the cellular immune response by affecting the mRNA stability of PD-L1 and showed the critical role of m6A in this process. These results suggest that we should pay attention to the role of METTL7B in the occurrence and development of glioma, especially in T cells apoptosis and immune response. In addition, we should also consider the unique correlation between METTL7B and the widely identified m6A participants in gliomas. Considering all the factors, we still need to carry out a large number of *in vitro* experiments to clarify the function of this molecule in gliomas.

## Data Availability Statement

The datasets presented in this study can be found in online repositories. The names of the repository/repositories and accession number(s) can be found in the article/**Supplementary Material**.

## Author Contributions

CL, SW, WX, SH, NX and YZ contributed to conception and design of the study. CL and SW organized the database. WS, SH provides methodological support. CL and SW performed the statistical analysis. QX and TY visualized the project. CL wrote the first draft of the manuscript. WS, WL, SZ, and YW wrote sections of the manuscript. All authors contributed to manuscript revision, read, and approved the submitted version.

## Funding

This work was supported by the basic research fund of Shenzhen (JCYJ20170405103953336).

## Conflict of Interest

Author SH was employed by the company Shenzhen Combined Biotech Co., Ltd.

The remaining authors declare that the research was conducted in the absence of any commercial or financial relationships that could be construed as a potential conflict of interest.

The authors declare that this study received funding from the basic research fund of Shenzhen (JCYJ20170405103953336). The funder was not involved in the study design, collection, analysis, interpretation of data, the writing of this article or the decision to submit it for publication.

## Publisher’s Note

All claims expressed in this article are solely those of the authors and do not necessarily represent those of their affiliated organizations, or those of the publisher, the editors and the reviewers. Any product that may be evaluated in this article, or claim that may be made by its manufacturer, is not guaranteed or endorsed by the publisher.

## Supplementary Material

Supplementary Figure S1 Survival analysis of cases in TCGA training cohort stratified by gender, WHO grades and Radiotherapy. Kaplan-Meier curves of OS in **(A)** male, **(B)** female, **(C)** G2, **(D)** G3, **(E)** with or **(F)** without radiotherapy patients based on risk score in low grade glioma population.

Supplementary Figure S2 Survival analysis of cases in TCGA validation cohort stratified by gender, WHO grades and Radiotherapy. Kaplan-Meier curves of OS in **(A)** male, **(B)** female, **(C)** G2, **(D)** G3, **(E)** with or **(F)** without radiotherapy patients based on risk score in low grade glioma population.

Supplementary Figure S3 Validation the prognostic performance of patients carrying wild‐type IDH1 genes, 1p19q codeletion or MGMT methylation. **(A)** IDH1 wild-type (WT) and mutation (Mutant) proportions in High Risk or Low Risk. **(B)** Chromosome 1p19q codeletion or not (Non) proportions in different risk groups. **(C)** MGMT methylation or not proportions in different risk groups. Kaplan-Meier curves of OS in IDH1 WT **(D)**, IDH1 mutant **(E)**, 1p19q codeletion **(F)**, 1p19q non-codeletion **(G)**, MGMT methylation **(H)** and MGMT unmethylation **(I)** based on risk score.

Supplementary Figure S4 Construction of weighted risk stratification gene co-expression network and functional enrichment analysis. **(A)** The scale-free fit index for soft thresholding powers. **(B)** Selecting the soft threshold of 4, checking the node connection number, and verifying the network connectivity of a scale-free network. **(C)** The correlation between network modules.

Supplementary Figure S5 Functional enrichment analysis of co-expression networks which containing risk stratification genes. The co-expression module of weighted gene and the Gene Ontology Analysis of all the module gene within **(A)** MLLT3, FAM66C, **(B)** SEL1L3, SOX13, **(C)**EFHB colored by specific cluster ID.

Supplementary Figure S6 Correlation between marker genes and risk grading genes. Marker genes related to **(A)** extracellular matrix remodeling, **(B)** immune response, **(C)** cytokine, and **(D)** chemokine release were selected from MsigDB database, and the correlation between the above marker genes and risk grading genes was analyzed in TCGA-LGG data set.
