# Supplementary figures and images for: A Whole Exon Screening-Based Score Model Predicts Prognosis and Immune Checkpoint Inhibitor Therapy Effects in Low-Grade Glioma
Source: Front Immunol. 2022 Jun 13;13:909189. doi: 10.3389/fimmu.2022.909189 (PMC9234137; doi:10.3389/fimmu.2022.909189)

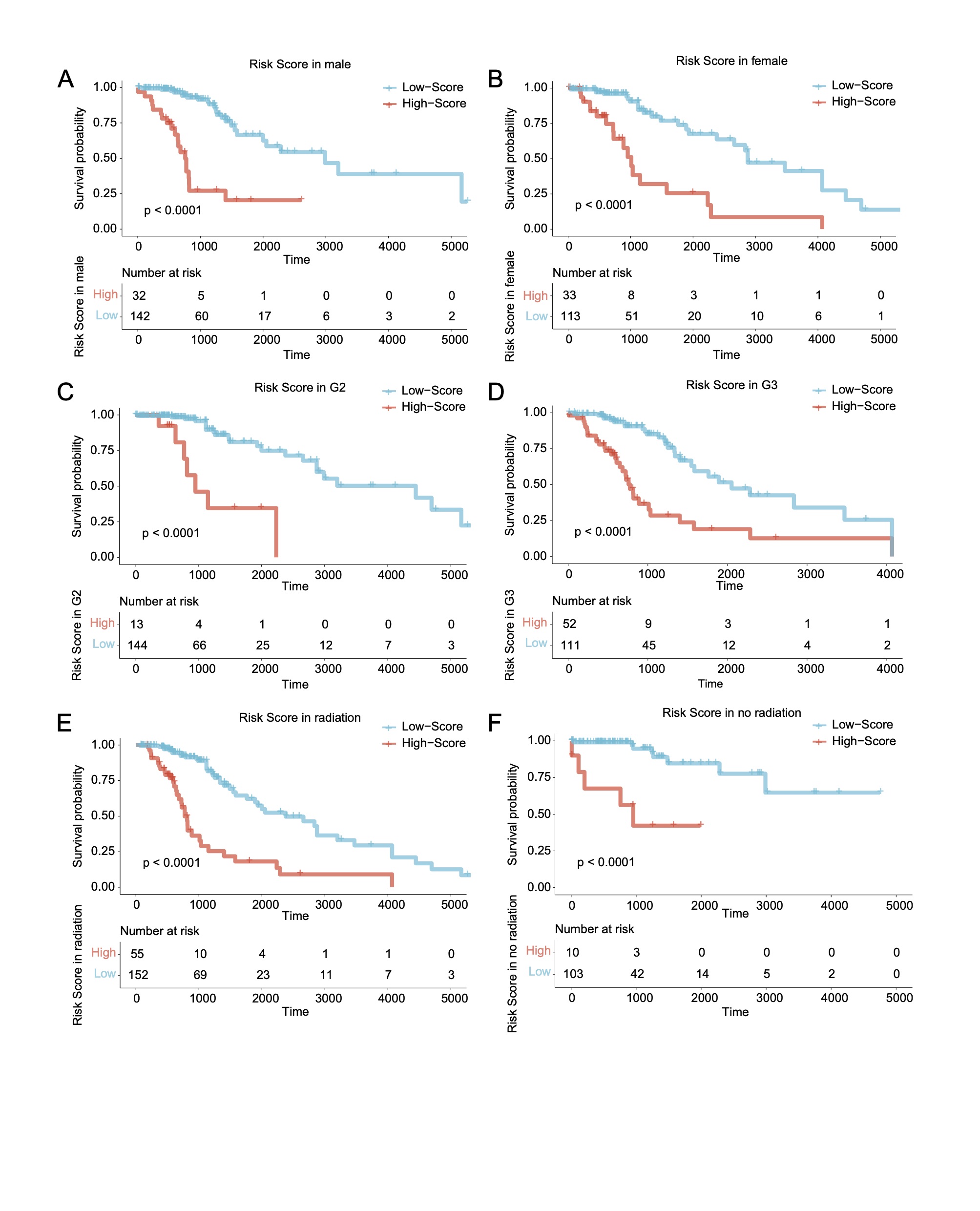

Supplement: Supplementary Figure S1 [file Image_1.jpeg]

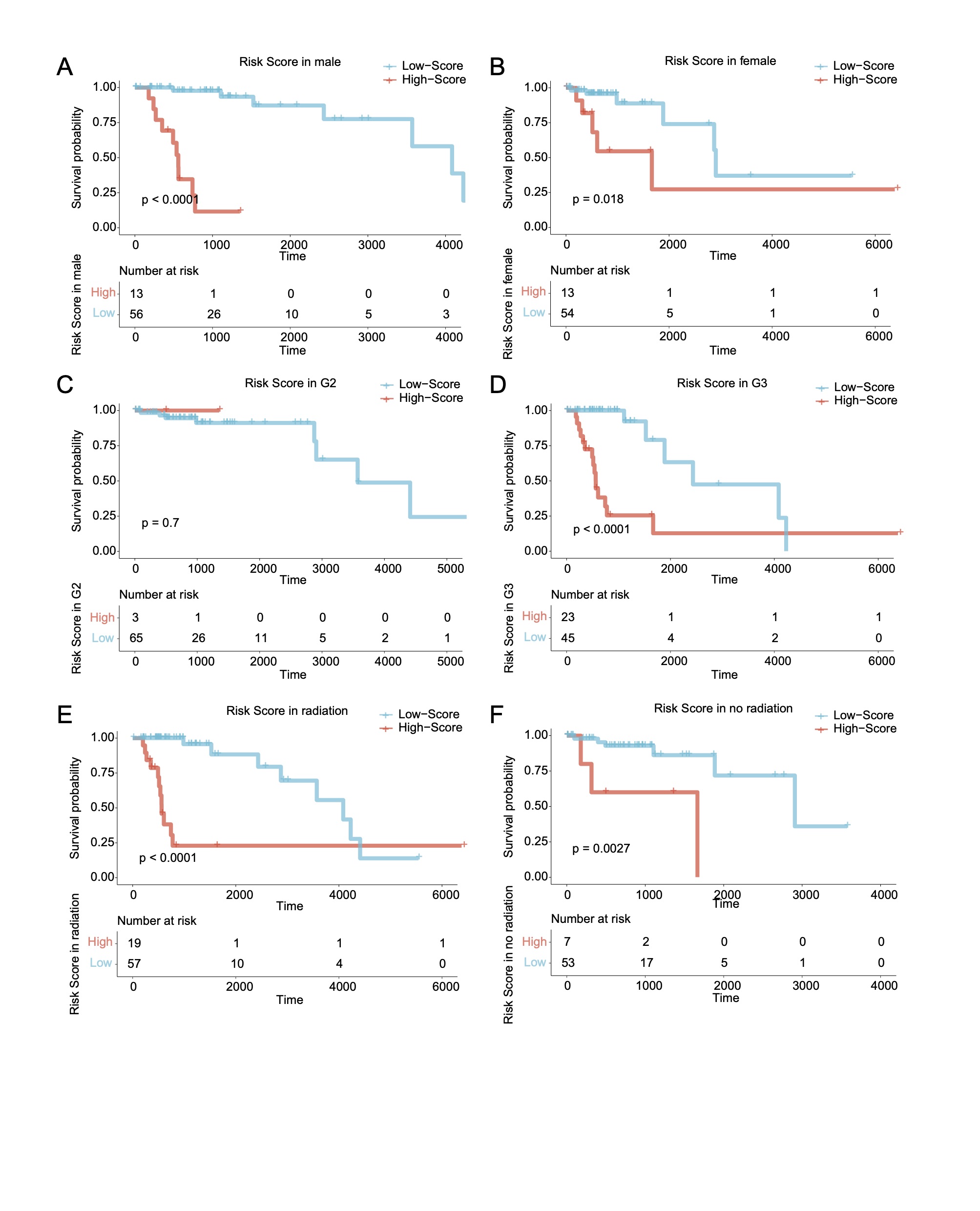

Supplement: Supplementary Figure S2 [file Image_2.jpeg]

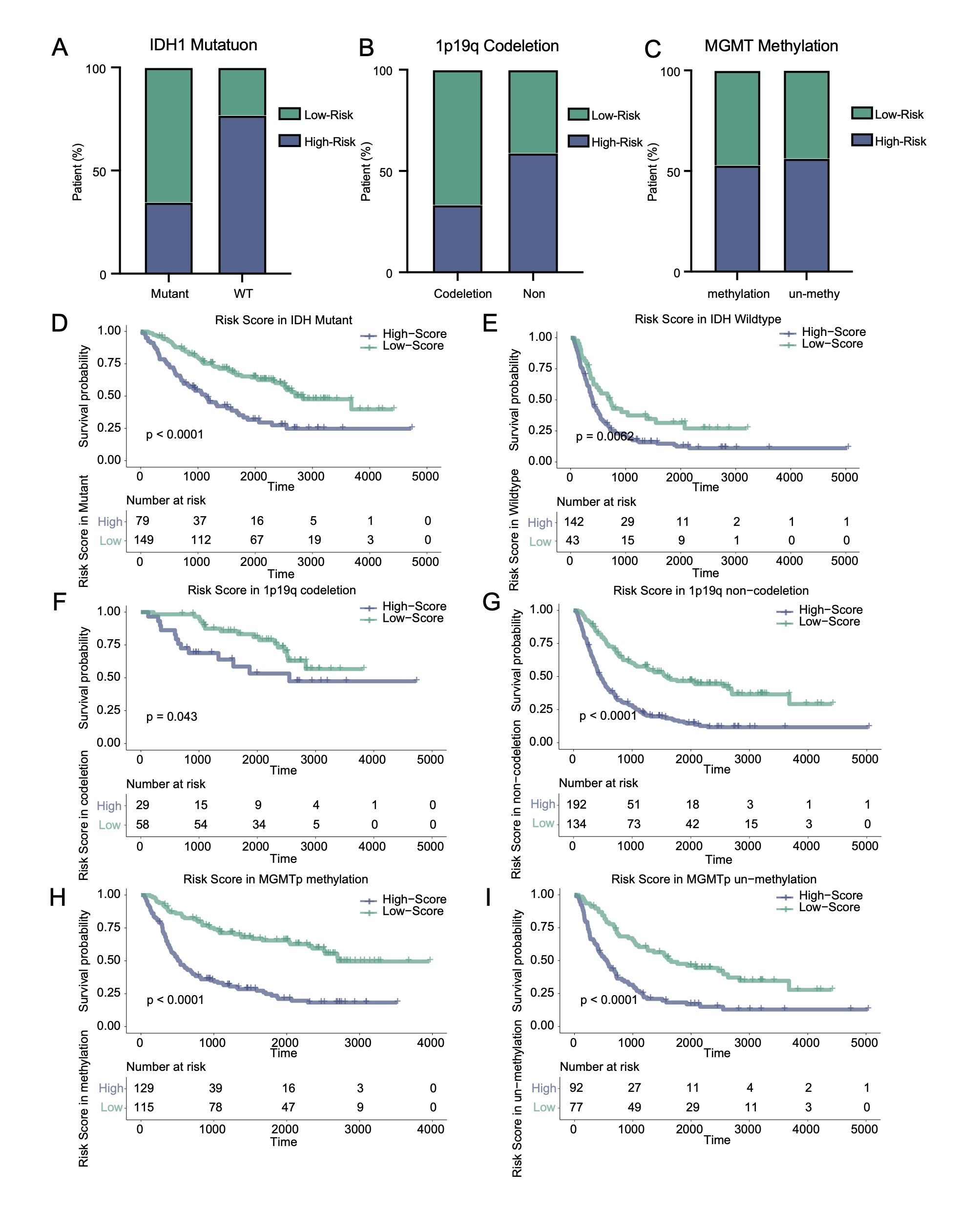

Supplement: Supplementary Figure S3 [file Image_3.jpeg]

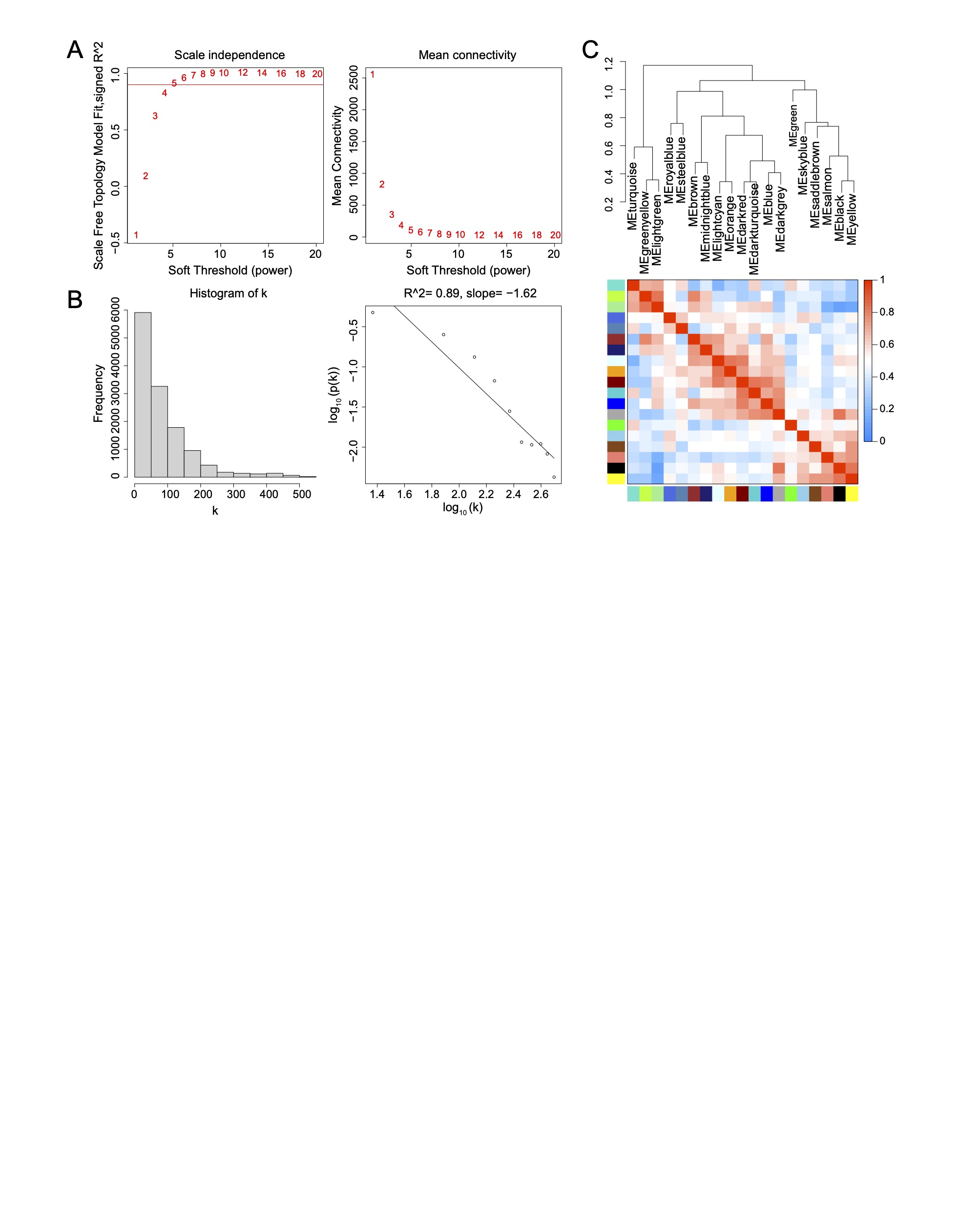

Supplement: Supplementary Figure S4 [file Image_4.jpeg]

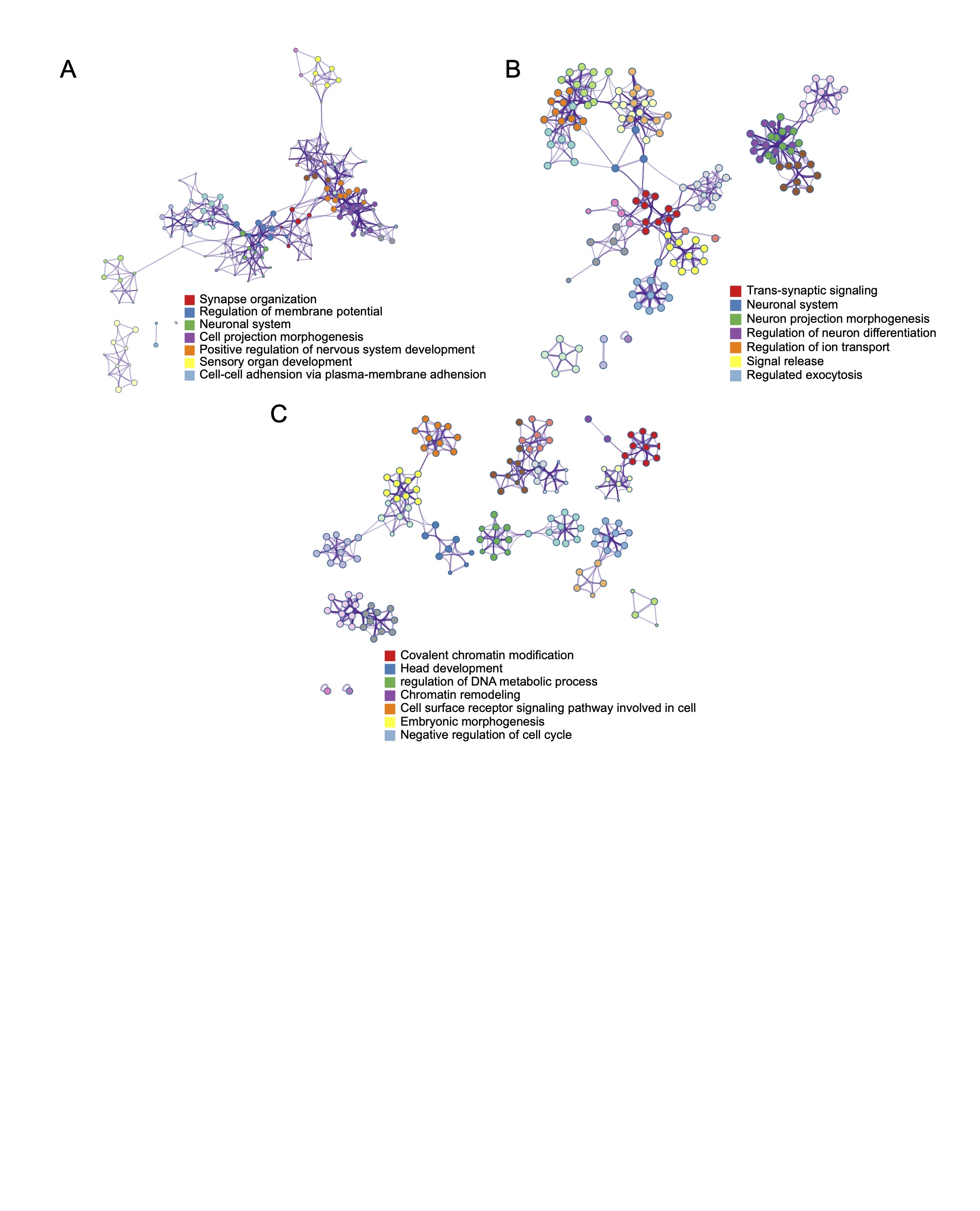

Supplement: Supplementary Figure S5 [file Image_5.jpeg]

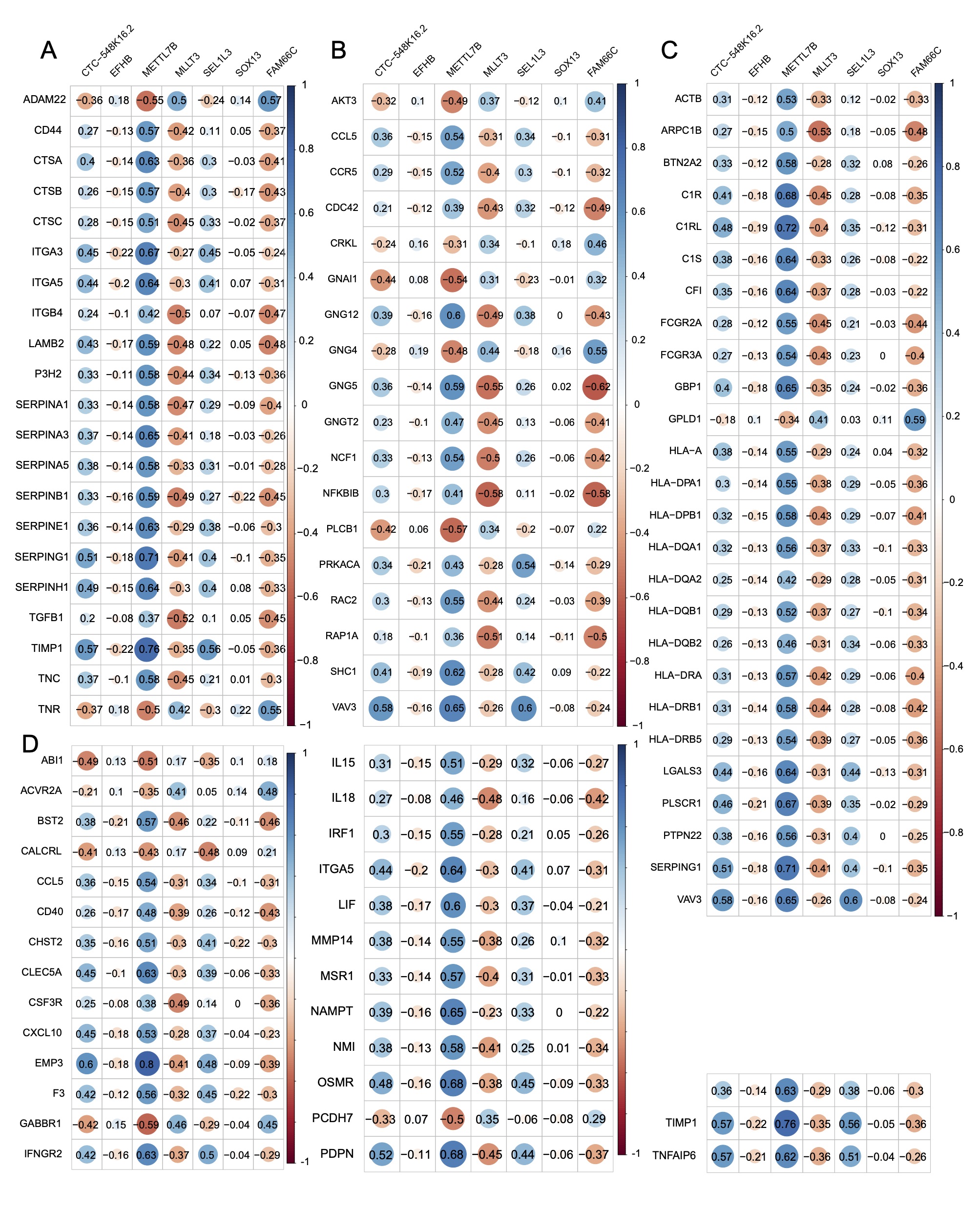

Supplement: Supplementary Figure S6 [file Image_6.jpeg]
