# Supplementary material for: A Whole Exon Screening-Based Score Model Predicts Prognosis and Immune Checkpoint Inhibitor Therapy Effects in Low-Grade Glioma
Source: Front Immunol. 2022 Jun 13;13:909189. doi: 10.3389/fimmu.2022.909189 (PMC9234137; doi:10.3389/fimmu.2022.909189)
Supplement: Supplementary file 7 [file DataSheet_1.docx]

**Supplementary Data:**

**Table 1**. Seed sequence for siRNA, shRNA-lentivirus.

| Name | Seed Sequence (5’ to 3’) |
| --- | --- |
| siMETTL7B-1# | CGGGAGCUCUUCAGCCAGA |
| siMETTL7B-2# | GGAAGGUCCUGCAGGAGGU |
| shMETTL7B-263 | GGAGCCAACTTTCAGTTCTAC |
| shMETTL7B-461 | GGCTGATGGCTCCATGGATGT |

**Table 2**. Primers for quantitative RT-PCR

| Primer name | Property | Sequence (5’ to 3’) |
| --- | --- | --- |
| CD274 | Forward primer | TGGCATTTGCTGAACGCATTT |
|  | Reverse primer | TGCAGCCAGGTCTAATTGTTTT |
| PD-L2 | Forward primer | ACCCTGGAATGCAACTTTGAC |
|  | Reverse primer | AAGTGGCTCTTTCACGGTGTG |
| METTL7B | Forward primer | ATGTGGTGGTCTGCACTCTG |
|  | Reverse primer | ATTTCGGAGAACTGGGCGTT |
| ACTIN | Forward primer | CATGTACGTTGCTATCCAGGC |
|  | Reverse primer | CTCCTTAATGTCACGCACGAT |
